# *Ampiang-Dadih-*a combination of Indonesian traditional fermented buffalo milk and black glutinous rice–prevents hypercholesterolemia and liver cell degeneration *in vivo*: A pilot study

**DOI:** 10.5455/javar.2024.k773

**Published:** 2024-06-05

**Authors:** Sri Rahmatul Laila, Eddy Sukmawinata, Falsa Martiana Kencana Putri, Ilham Akbar, Luthfiyyah Fitri Zahra, Srihadi Agungpriyono, Umi Cahyaningsih, Tutik Wresdiyati

**Affiliations:** 1 Division of Anatomy, Histology, and Embryology, School of Veterinary Medicine and Biomedical Science, IPB University, Bogor, Indonesia; 2 Research Centre for Veterinary Science, Research Organization for Health, National Research and Innovation Agency, Bogor, Indonesia; 3 Undergraduate of Veterinary Medicine, School of Veterinary Medicine and Biomedical Science, IPB University, Bogor, Indonesia; 4 Division of Parasitology and Medical Entomology, School of Veterinary Medicine and Biomedical Science, IPB University, Bogor, Indonesia

**Keywords:** *Ampiang*, *Dadih*, hypercholesterolemia, rat model, traditional yogurt

## Abstract

**Objective:**

We aimed to evaluate the potential of *Ampiang-Dadih *(AD), a combination of Indonesian traditional fermented buffalo milk (*Dadih*) and black glutinous rice flakes (*Ampiang*) as an anti-hypercholesterolemic agent and to prevent liver-cell degeneration using a rat model.

**Materials and Methods:**

A mixture of black glutinous rice powder (0.3 gm/gm feed) and fermented buffalo milk (3.74/200 gm BW) was prepared to obtain AD. Fifteen adult male Sprague Dawley rats were divided into three groups of five animals each: (A) negative control group (distilled water; 5 weeks), (B) hypercholesterolemia group (1% cholesterol per feed; 5 weeks), and (C) preventive AD group (1% cholesterol and AD; 5 weeks). The blood lipid profiles were measured at weeks 2, 4, and 5. The liver enzyme activity, cholesterol level, and histology were observed at the end of week 5.

**Results:**

AD administration simultaneously with cholesterol in Group C significantly prevented an increase in total plasma cholesterol and low-density lipoprotein levels compared to Group B. Alanine transaminase and aspartate transaminase were maintained at normal levels in Group C. Furthermore, the levels of liver cholesterol and liver cell degeneration in Group C were also maintained because of AD administration compared to that in Group B.

**Conclusion:**

This study demonstrated that AD has the potential to be developed as a functional food for hypercholesterolemia prevention.

## Introduction

Cardiovascular diseases are associated with the heart and blood vessels. According to the World Health Organization, in 2019, almost 18 million people died from cardiovascular diseases. Developing countries, including countries in Southeast Asia, account for 75% of these deaths [1]. In Indonesia, the highest number of fatalities are caused by cardiovascular disease, which accounts for 37% of all deaths, and these deaths are mainly caused by hypercholesterolemia [2]. A health survey conducted in 2018 identified five provinces in Indonesia with the highest prevalence of cardiovascular disease: North Kalimantan (2.2%), Gorontalo (2.0%), Yogyakarta (2.0%), Aceh (1.6%), and West Sumatera (1.6%). In particular, the consumption of a high-fat diet, including a high-cholesterol diet, more than once per day was reported by 38.1% of people in the West Sumatra Province [3]. Hypercholesterolemia can occur due to a high cholesterol intake and cholesterol synthesis in the liver [4]. In contrast, the West Sumatra Province is rich in healthy traditional foods such as fermented buffalo milk products that display probiotic activity exhibiting benefits in preventing hypercholesterolemia [5].

High blood cholesterol levels can be prevented and reduced through the consumption of drugs, herbs, and functional foods. The association between food and health has become a central focus for several consumers, leading to more informed and health-conscious choices. The market for dietary supplements and functional foods has grown as consumers seek products that offer specific health benefits, such as probiotics for gut health and increase immune system [6]. *Dadih*, West Sumatra’s (Indonesia) traditional fermented buffalo milk, contains various types of lactic acid bacteria (LAB), which have been reported to exhibit bile salt hydrolysis activity, deconjugate taurocholate, and bind to cholesterol *in vitro *[7]. *Dadih *consumption reduced cholesterol levels in alloxan-induced diabetes rats [8]. Interestingly, locals usually consume *Dadih* in various combinations; the most common combination is with *Ampiang*, which is called *Ampiang*-*Dadih* (AD) [9]. *Ampiang* is made from black glutinous rice (*Oryza sativa* var. glutinosa L), heated, and pounded flat (flake). Methanol extracts from black glutinous rice at a concentration of 200 ppm *in vitro* can bind free cholesterol by up to 47.46% [10]. In mice, black glutinous rice administration reduced the expression of sterol regulatory element binding protein-2, acyl-coenzyme A cholesterol acyltransferase-2, and 3-hydroxyl-3-methylglutaryl coenzyme A in the liver, and had the potential to reduce blood cholesterol levels [11].

Therefore, the combination of AD can potentially be consumed as a functional food for hypercholesterolemia prevention, which may prevent liver cell damage. However, the benefits of *Ampiang* and *Dadih* are still poorly understood when consumed in combination. Therefore, this study evaluated the efficacy of AD administration to prevent hypercholesterolemia and liver-cell degeneration in rat models. Our findings will provide information and primary data for developing functional food products for cardiovascular health from local sources.

## Materials and Methods

### Ethical approval

This study received approval from the Animal Care and Use Committee of IPB University, Indonesia (No. 009/KEH/SKE/VI/2022).

### AD sample and preparation

*Ampiang* (black glutinous rice flakes) and *Dadih* (fermented buffalo milk) were collected from Solok Regency, West Sumatra, Indonesia. In brief, *Dadih* was prepared from swamp buffalo milk, which was fermented spontaneously in bamboo tubes without inoculating any starter culture, and incubated for four days at room temperature (25°C–27°C) to obtain a firm consistency. Subsequently, *Dadih* was refrigerated until further analysis. Ampiang was bought from a traditional market in Solok Regency. Subsequently, *Ampiang* was ground and sieved using an 80 mesh. AD was prepared by mixing Ampiang 0.3 gm/gm feed weight (modification from Park et al. [12]), *Dadih* 3.74/200 gm of body weight (BW), and distilled water, followed by homogenization. Commercial feed (Indofeed^®;^, Jakarta, Indonesia) was used as the standard feed. The nutritional contents of fresh *Dadih*, *Ampiang*, and commercial standard feed were obtained using proximate analyses as described in Table 1.

### Bacterial analysis

Bacterial identification was performed using LAB. Bacterial enumeration was performed based on previously described methods using de Man-Rogosa-Sharpe (MRS) Agar (Becton, Dickinson and Company, Le Pont de Claix, France) [13]. The agar plates were incubated at 27°C under anaerobic conditions for 48–72 h. The incubation temperature was determined based on the temperature conditions at the sampling location. Isolation was performed based on the morphology of a single colony of bacteria growing on MRS agar plates and identified at the species level using MALDI-TOF MS (Bruker, MA, US).

### Animals

In this study, we used 15 male Sprague-Dawley rats weighing 190–250 gm obtained from the Indonesian Agency of Drug and Food Control. Rats were acclimatized for 10 days and administered commercial standard feed containing 10% BW, anthelmintics, anti-ectoparasites, anti-protozoa, vitamins, and drinking water ad libitum. The rats were housed in cages (46 × 34 × 15 cm) with sawdust at the bottom. All rat cages were placed at the Laboratory Animal Management Unit, School of Veterinary Medicine and Biomedical Science, IPB University, equipped with air conditioning and lighting for 12 h per day. During the study, the animals were monitored by the attending vet at the facility.

**Table 1.** Nutrients present in fresh *Dadih*, *Ampiang*, and standard feed (commercial feed for rat/Indofeed^®;^).

Ingredient	Water	Ash	Fat	Protein	Fiber
	%				
*Dadih*	78.46 ± 0.16	0.93 ± 0.04	6.02 ± 0.24	6.93 ± 0.10	0.09 ± 0.01
*Ampiang*	9.77 ± 0.32	8.04 ± 0.13	4.34 ± 0.03	16.75 ± 0.11	7.17 ± 0.01
Standard feed	10.33 ± 0.05	1.88 ± 0.08	0.92 ± 0.07	17.46 ± 0.32	0.92 ± 0.08

**Figure 1.**
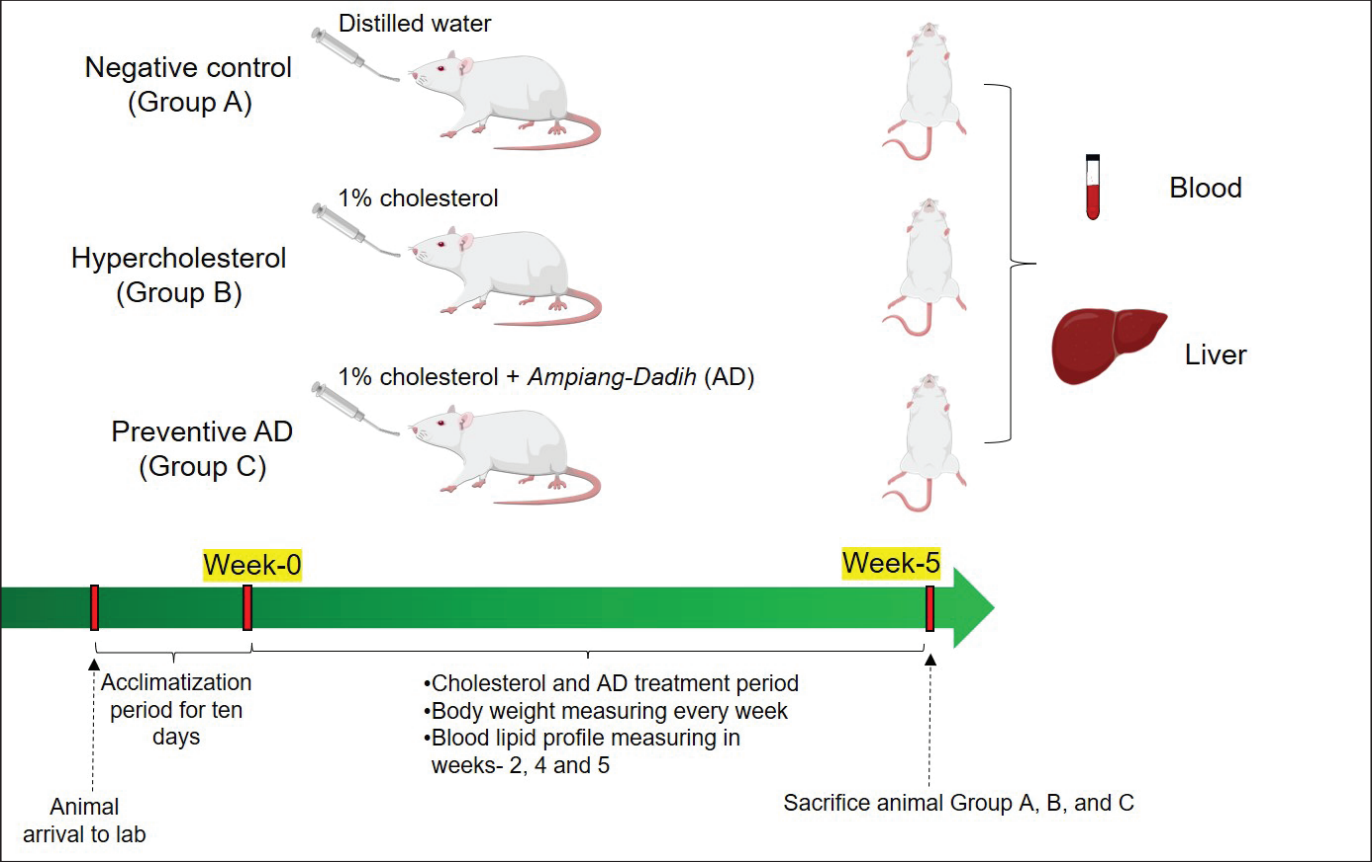
Group treatments in this study and sample collection. Group A, negative control; Group B, hypercholesterolemia group; Group C, preventive AD group.

To investigate the efficacy of AD in preventing hypercholesterolemia, rats were randomly divided into three groups: negative control (A), hypercholesterolemia (B), and preventive AD (C), with five rats in each group based on the Federer formula. The rats in the negative control group were only treated with distilled water. The hypercholesterolemia group (B) rats were administered daily via gastric sonde with a cholesterol solution made from cholesterol powder (FUJIFILM Wako, Japan) (1% of the feed weight, mixed with corn oil as a solvent). The efficacy of AD for hypercholesterolemia prevention was observed in group C with daily feedings of 1% cholesterol and AD simultaneously. The treatments for Groups A–C were conducted for five weeks, as visualized in Figure 1.

### BW and lipid profiles

The BW was measured weekly using a digital scale, and the blood lipid profiles were measured at weeks 2, 4, and 5. The blood samples were collected from the vena caudalis lateralis [14] using a sterile 20-gauge needle and placed in a plain vacutainer (Vaculab Plain 3 ml K3^®;^; OneMed). After coagulating and centrifuging the blood, serum was collected to analyze the total plasma cholesterol (TPC), high-density lipoprotein cholesterol (HDL-C), and triglycerides (TGs) using a spectrophotometer (Photometer 5010 RIELE™, Berlin, Germany). Low-density lipoprotein cholesterol (LDL-C) levels were calculated using the Friedewald formula.

### Liver enzyme activity and organ sampling

Euthanasia was performed after five weeks. Rats were fasted for 12 h before blood sampling and were then anesthetized using a combination of ketamine 70 mg/kg BW (Ket-A-100^®;^, Agrovet) and xylazine 10 mg/kg BW (Xyla^®;^, Interchemie) which was administered intraperitoneally. Blood samples were then collected intracardially and placed in a vacuum tube without anticoagulant to obtain the serum. From this serum, we analyzed the levels of TPC, HDL-C, TG, alanine transaminase (ALT), and aspartate transaminase (AST) as liver enzyme activity parameters using a spectrophotometer. After signs of death were detected, the abdominal and thoracic cavities were opened to collect the liver. One gram of liver was collected and lysed for organ cholesterol analysis (Cholesterol RM^®;^, Glory Diagnostic) using a spectrophotometer at a wavelength of 50 nm.

### Histological analysis

The remaining liver tissue was washed with physiological NaCl and placed in Bouin’s fixative solution. After 24 h, the mixture was transferred to 70% alcohol as the fixation stopping point. The dehydration stage began by adding 80% alcohol, 90% alcohol, and 95% alcohol sequentially for 24 h each, and then the samples were placed in absolute alcohol I, II, and III for 60 min each. Next, xylol was used for tissue clearing. The organ pieces were infiltrated and embedded in paraffin blocks. After cutting the block to a thickness of 4 mm, tissue slides were de-paraffinized, rehydrated, and stained with hematoxylin and eosin. The slides were then mounted and observed under a light microscope (Olympus CX31) with 400× magnification, and images were captured using a camera-equipped microscope (IndoMicro HDMI Camera + Adapter Exfocus-0.5×). The condition of the liver, including cells with lipid degeneration, Kupffer cells, and liver tissue density, was determined in each group using ImageJ software.

### Data analysis

The nutritional ingredients are presented in tabular form, and the LAB data are described in the text. The Kolmogorov-Smirnov test was performed to check for the normality of the data. The significance of the observed parameters was determined using SPSS Statistics ver. 25 (IBM) and visualized using GraphPad Prism 7 X LM. The average BW, blood lipid profiles, liver enzyme activity, liver cholesterol content, and liver tissue density were analyzed using a one-way analysis of variance (*p*-value 0.05) and Tukey’s post-hoc test to observe group differences. The histological condition of the liver is shown as a photomicrograph, and then the cells with lipid degeneration and Kupffer cells are analyzed.

## RESULTS

### Nutritional ingredients and LAB population

The nutritional ingredients of each group are presented in Table 2. The commercial standard feed had a higher carbohydrate content than feed supplemented with AD (*p *< 0.01). AD administration increased protein levels (*p *= 0.02), fat content, and crude fiber content (*p *< 0.01) compared to that in the commercial standard feed. Bacterial composition revealed that a yield of 5.4′10^−7^ colony-forming units of LAB was obtained from the *Dadih* sample. A total of 55 LAB isolates were identified from 83 isolates. *Lactobacillus plantarum* was frequently identified as the dominant species (69.1%; 38/55), followed by *Levilactobacillus brevis* (25.5%; 14/55) and *Lacticaseibacillus paracasei* (5.4%; 3/55).

### BW and lipid profiles

**Figure 2.**
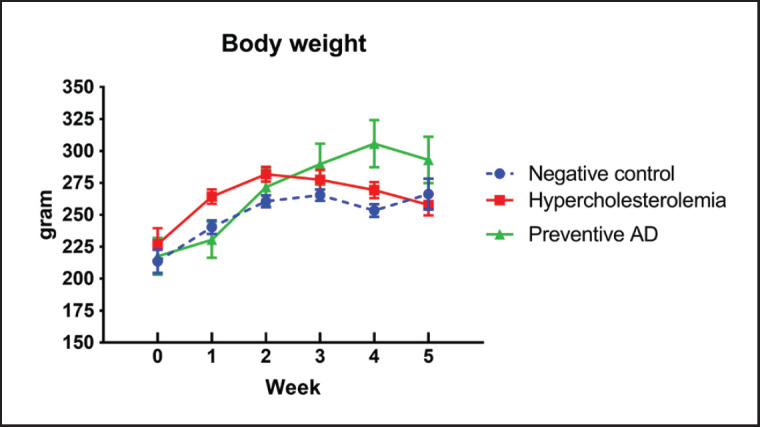
Average BW of rats in each treatment group throughout the 5 weeks study period. No significant differences exist in the BW at baseline (week-0). The BW of the hypercholesterolemia group (B) significantly increased in weeks 1 and 2 and then decreased. The BW of the preventive AD group significantly increased at weeks 4 and 5 compared to that of the control group. AD = *Ampiang-Dadih.*

**Figure 3.**
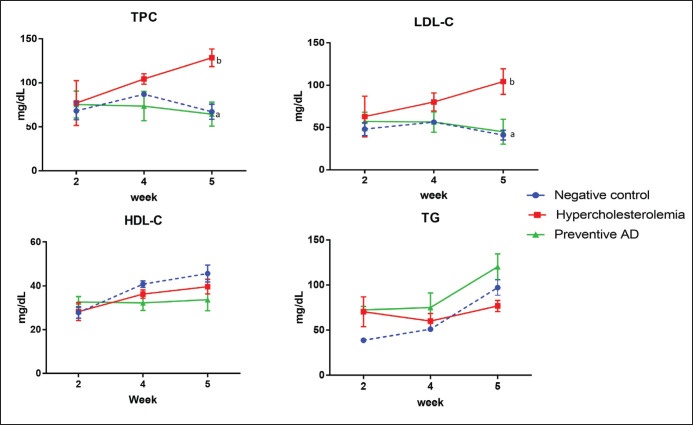
Blood lipid profiles in each group of experimental rats. TPC, LDL-C, HDL-C, and TG levels were measured in each group of experimental rats during the five weeks of treatment. Different superscripts indicate significant differences between groups (*p *< 0.05). AD prevented an increase in TPC and LDL and did not affect HDL and TG levels. AD, *Ampiang-Dadih*; TPC, Total plasma cholesterol; LDL-C, low-density lipoprotein cholesterol; HDL-C, high-density lipoprotein cholesterol; TG, triglyceride.

No differences were observed in the BW between the groups at week 0. From the first to the second week, the BW of the rats increased in all groups (Fig. 2). Subsequently, the rat BW in the hypercholesterolemia group (B) exhibited a decreasing trend in the third, fourth, and fifth weeks, whereas the BW of the negative control (A) and preventive AD groups (C) fluctuated slightly. At the end of week 5, no significant difference was observed in the BW between the groups (*p *= 0.190). Figure 3 shows that the levels of TPC and LDL in preventive AD groups (C) were significantly lower than those in the hypercholesterolemia group (B) (*p *< 0.01) and control group (A). Nevertheless, administering AD did not significantly affect HDL (*p *= 0.775) or TG levels (*p *= 0.280) in rats in this study.

### Liver enzyme activity and cholesterol organ

**Figure 4.**
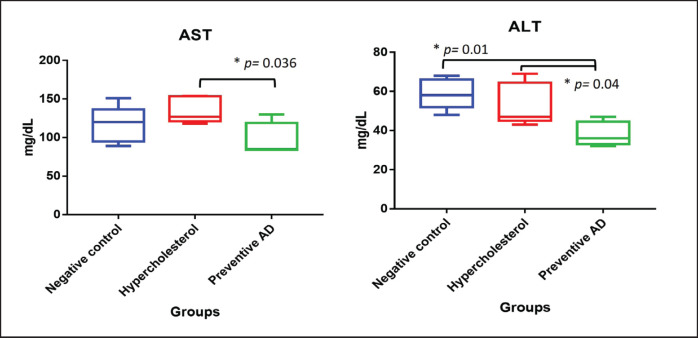
AST and ALT profiles of all experimental groups. Hypercholesterolemia increased AST levels in rats, whereas AD maintained AST levels similar to those in the negative control group. Rats treated with AD to prevent hypercholesterolemia had the lowest ALT levels. AD = *Ampiang-Dadih.*

**Figure 5.**
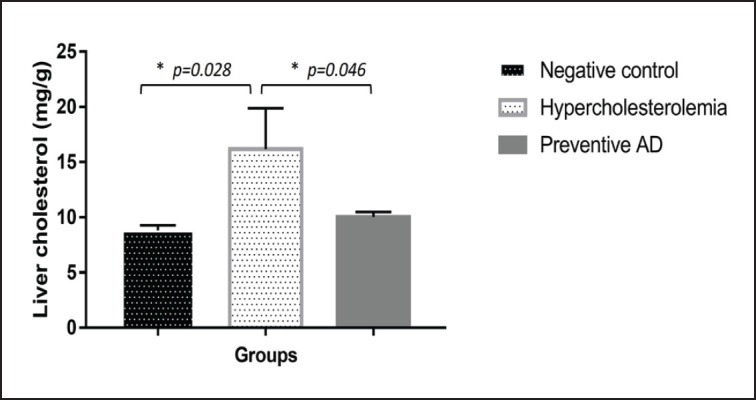
Liver organ cholesterol levels in each experimental group**.** The liver cholesterol content in the hypercholesterolemia group was higher than that in the other groups. Rats treated with AD had the same liver cholesterol levels as those in the negative control group (*p *> 0.05). AD = *Ampiang-Dadih.*

Rats in the hypercholesterolemia group (B) experienced an increase in AST levels (Fig. 4). AD, as a hypercholesterolemia prevention agent, could maintain normal AST levels. The lowest ALT level was observed in Group C. The ALT levels in the hypercholesterolemia group (Group B) were not significantly altered compared with those in the negative control (A) (*p *= 0.56). The hypercholesterolemia group (Group B) had the highest liver cholesterol content (*p *< 0.05), whereas the preventive AD group (Group C) had the same liver cholesterol level as the negative control group (Group A) (Fig. 5).

**Table 2.** Nutrient content in each rat group per day based on 100% feed dry weight.

Group	Feed	Ash	Protein	Fat	Carbohydrate	Fiber	Giving 1% cholesterol
		%					
A	Commercial Standard feed	2.09 ± 1.32^a^	18.91 ± 0.30^a^	1.02 ± 0.10^a^	76.72 ± 0.09^b^	1.03 ± 0.12^a^	No
B	Commercial Standard Feed and Cholesterol	2.14 ± 0.05^a^	18.60 ± 0.14^a^	1.05 ± 0.07^a^	76.59 ± 0.27^b^	0.96 ± 0.04^a^	Yes
C	Combination of *Ampiang-dadiah* and standard feed	4.56 ± 0.09^b^	21.49 ± 0.16^b^	7.21 ± 0.22^b^	62.94 ± 0.13^a^	2.92 ± 0.01^b^	Yes

The different superscripts for nutrient content indicate a significant difference among groups (*p *< 0.05).

### Liver histology

**Figure 6.**
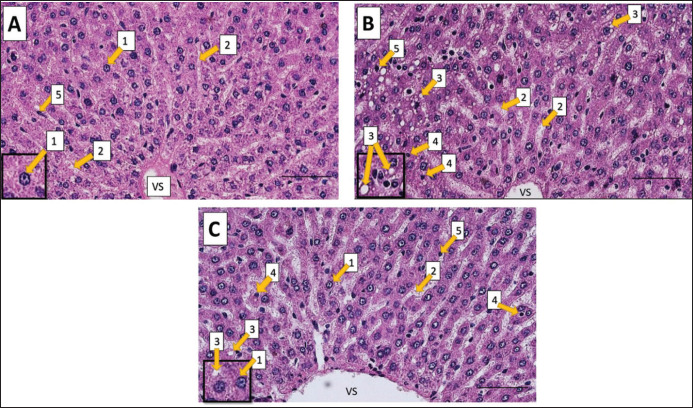
Histological liver images of treated rats after five weeks of study. The liver of negative control rats (A), hypercholesterolemia rats (B), and preventive AD rats (C). 1: normal liver cells; 2: sinusoids; 3: fatty liver (lipid degeneration); 4: necrosis; 5: Kupffer cells. Line bars represent 50 µm. AD = *Ampiang-Dadih*, VS = Vena centralis.

Rat livers from the negative control group showed that the liver cells were still in a normal condition, with a tight and regular arrangement of liver cell parenchyma and normal sinusoids (Fig. 6A). In the hypercholesterolemia group, liver cells appeared to experience sinusoidal expansion, lipid degeneration (fatty liver), and necrosis (Fig. 6B). Livers in the preventive AD group (Group C) showed that most liver cells were still in the normal condition, with slight sinusoidal expansion (Fig. 6C). The percentage of cell degeneration, necrosis, Kupffer cells, and liver cell density in the preventive AD group were higher than those in the hypercholesterolemia group, as shown in Table 3.

## Discussion

In this study, rats administered 1% cholesterol and AD showed simultaneous increases in BW due to the high protein and fat content of AD [15]. Nevertheless, the total cholesterol plasma and LDL levels in the preventive AD group did not increase as much as in the hypercholesterolemia group. We assumed that this beneficial effect was associated with high leucine, isoleucine, and valine levels in buffalo milk [16]. Leucine increases the absorption of proteins present in AD under acidic conditions and could stimulate muscle protein synthesis instead of fat synthesis in rats [17].

Black glutinous rice, which is used to prepare *Ampiang*, contains a pigment called anthocyanin, which can reduce cholesterol levels by inhibiting lipid absorption and cholesterol synthesis [18]. Amrinola et al. [19] reported that black glutinous rice had a high fiber content. Fiber can bind cholesterol in the gut and prevent its absorption into the bloodstream. Furthermore, soluble fiber can bind with bile salts and disturb the digestion of lipids and cholesterol [20]. Thus, the combination of *Ampiang *and *Dadih* can help maintain a good blood lipid profile.

The liver is the primary organ involved in cholesterol metabolism. Inhibition of cholesterol absorption in the prevention AD group suggested that it could decrease liver cholesterol levels in rats. The liver responds to cholesterol intake from food and facilitates the release and metabolism of lipoprotein particles [21]. Lower ALT and AST levels in the preventive AD group indicated that AD could prevent cholesterol deposition and liver-cell degeneration. We detected various LABs in *Dadih*. Lactobacilli are probiotic bacteria that can be used as prophylactic or preventive agents and to lower cholesterol levels [22,23]. The *Dadih *sample in this study was dominated by *L. plantarum*. This bacterium has been reported to be capable of removing cholesterol *in vitro* [24]. Another study suggested that *Dadih* contains *L. lactis*, which might prevent hypercholesterolemia in rats [25]. While LAB may have some potential benefits related to fatty liver, it should not be considered a standalone or guaranteed solution. In addition to probiotics, peptides are found in fermented milk, including *Dadih *[26]. Peptides from casein can inhibit cholesterol absorption in intestinal epithelial cells [27].

High AST and ALT levels can lead to hypercholesterolemia and various health problems, including liver cell injury [28]. Moreover, long-term high cholesterol levels can cause oxidative stress and trigger histological changes in the liver [29]. In addition, high cholesterol levels can induce the development of non-alcoholic fatty liver disease, such as fatty changes in the liver with fibrosis, fatty degeneration, and steatosis [30]. Interestingly, AD consumption showed efficacy in protecting the rat livers from lipid degeneration and necrosis. Our findings suggest that AD consumption as a part of a healthy diet and lifestyle can potentially prevent hypercholesterolemia. This study was limited to the entire AD component and short-term hypercholesterolemia. Future studies are required to search for the best ingredients and formulas, confirm specific potential LABs, and assess their effect on long-term hypercholesterolemia in animal models and curative/lower plasma cholesterol levels *in vivo.*

**Table 3.** The percentage of liver cell degeneration, necrosis, Kupffer cells, and liver cell density.

Parameter	Group		
	A	B	C
Fatty liver/lipid degeneration	-	36%	5%
Necrosis	-	11%	5%
Kupffer cell	7%	16%	9%
Liver tissue density	95.75 ± 0.97^a^	87.42 ± 4.39^b^	94.69 ± 1.38^a^

The different superscripts for liver tissue density indicate a significant difference among groups (*p* < 0.05). A, negative control; B, hypercholesterolemia; C, preventive AD.

## Conclusion

AD could prevent hypercholesterolemia, maintain AST and ALT at normal levels, and protect liver cells from degenerative conditions when given simultaneously with 1% cholesterol induction in rat models. Our preliminary *in vivo* study suggests that AD has the potential to be developed as a functional food for anti-hypercholesterolemia from a local source. Moreover, our study can provide more information to local people about the health benefits of AD consumption.

## List of Abbreviations

AD, *Ampiang*-*Dadih*; ALT, alanine transaminase; AST, aspartate transaminase; BW, body weight; LAB, lactic acid bacteria; HDL-C, high-density lipoprotein cholesterol; LDL-C, low-density lipoprotein cholesterol; TG, triglycerides; TPC, total plasma cholesterol; VS, vena centralis.
